# β-Carotene-inulin colloidal particles coated with lactoferrin for food fortification

**DOI:** 10.1016/j.fochx.2026.104212

**Published:** 2026-07-14

**Authors:** Inga Gabriunaite, Tatjana Kavleiskaja, Rūta Gruškienė, Jolanta Sereikaitė

**Affiliations:** a Department of Chemistry and Bioengineering, Faculty of Fundamental Sciences, Vilnius Gediminas Technical University, Sauletekio av. 11, LT-10223 Vilnius, Lithuania; b Institute of Chemistry, Faculty of Chemistry and Geosciences, Vilnius University, Naugarduko str. 24, LT-03225 Vilnius, Lithuania

**Keywords:** β-Carotene, Inulin, Lactoferrin, Complexation, Antioxidant activity, Fortification of yogurt and beverages

## Abstract

β-Carotene exhibits antioxidant properties. However, β-carotene degrades under the action of oxygen, heat, and light and is insoluble in water. This study aimed to develop a new β-carotene formulation using inulin and lactoferrin. β-Carotene-loaded inulin complexes were coated with lactoferrin at pH values of 4, 6, and 8. The obtained particles were dispersible in water in the range of 17.4–22.9 mg/mL. The hydrodynamic diameter of particles was 250–300 nm or greater depending on the [CAR-IN]/[LF] ratio and the pH value. Encapsulated β-carotene retained its antioxidant activity. The new formulation was studied in real food systems. The kinetic parameters of the colour changes in the beverages and yogurt fortified with the new particles were determined under various storage conditions. At 4 °C, the reaction rate constants (d^−1^) of changes in chroma were 0.07 ± 0.01, 0.09 ± 0.02 and 0.11 ± 0.01 for birch sap, mineral water and natural yogurt, respectively.

## 1 Introduction

People consume a lot of fast food due to their fast-paced lifestyles (Hoff-Jorgensen & Leer, 2025; Sarhan et al., 2025). The human diet does not contain enough fresh fruits and vegetables and consequently lacks various micronutrients, such as antioxidants, that provide health benefits. The development of functional foods could overcome these limitations. The global functional foods market is currently growing, reaching more than 272 billion dollars in 2021 (Guo, 2025). β-Carotene (CAR) is a well-known antioxidant found in various colourful fruits and vegetables, such as carrots, pumpkins, apricots, and others (Melendez-Martinez et al., 2022). In addition, CAR is a precursor of vitamin A. In human organisms, the enzyme β, β-carotene 15,15′–monooxygenase 1 catalyses the cleavage of CAR into two molecules of all-trans-retinal. Furthermore, the enzyme retinal reductase can reduce retinal to retinol (Melendez-Martinez, 2019). The insolubility of CAR in water and its sensitivity to light, oxygen, and temperature reduce the possibilities of CAR application in functional foods. The encapsulation approach is usually used to protect CAR from environmental damage. Since CAR is oil-soluble, the most popular carriers are lipid-based, such as emulsions, liposomes, solid lipid nanoparticles, and nanostructured lipid carriers (Rehman et al., 2020). However, there are some limitations to the use of these lipid-based delivery systems in water-based foods. Their thermodynamic and physical instability can lead to particle aggregation and phase separation. Furthermore, chemical degradation can occur as a result of oxidation reactions (Laein et al., 2024). Recently, water-dispersible CAR complexes with chitooligosaccharides (Bockuviene & Sereikaite, 2019), xylan (Straksys et al., 2022) or more complexed particles based on xylan, chitooligosaccharides and fucoidan (Straksys et al., 2024) have been developed. Therefore, studies on new formulations of this carotenoid are important to extend its use in functional foods.

Lactoferrin (LF) is an iron-binding glycoprotein with a molecular mass in the range of 77–91 kDa. In mammals, it is found mainly in milk. It is also present in other secretory fluids, such as saliva, tears, and bronchial mucus. There is a high homology of protein sequences between species. The sequence homology between human and bovine lactoferrin is approximately 78%. (Fijalkowski et al., 2025; Kim et al., 2025). Lactoferrin is a globular protein and belongs to the transferrin family. Bovine lactoferrin is composed of 689 amino acid residues, and its polypeptide chain folds into two symmetric lobes named the N-lobe (residues 1–341) and the C-lobe (residues 342–689) which are connected by a three-turn α-helix (Dyrda-Terniuk & Pomastowski, 2023). Lactoferrin exhibits a variety of biological properties. Not only does it deliver iron ions, it also exhibits antimicrobial, antioxidant, antiviral and immunomodulatory activities. Due to its multifaceted roles, it shows great potential for use as a nutritional additive in the formulation of functional foods and the fortification of infant formula (Demir et al., 2025; Dyrda-Terniuk & Pomastowski, 2023; Kim et al., 2025). Furthermore, recent investigations have demonstrated that lactoferrin can form complexes with bioactive molecules such as polyphenols and chemotherapeutic drugs (Chen et al., 2023; Sabra & Agwa, 2020). This functionalisation of lactoferrin extends its potential use in the food industry and in nanomedicine.

Inulin (IN) is a linear polysaccharide composed of fructofuranosyl units linked by β-(2 → 1) glycosidic bonds. The degree of polymerisation of inulin molecules is 2–60. They usually terminate with a glucose residue linked by a sucrose-type linkage. Inulin molecules with a degree of polymerisation of no more than 10 are called oligofructose (Mensink et al., 2015). As the β-(2 → 1) glycosidic bond is indigestible to the human digestive system, inulin is a soluble fibre and serves as a prebiotic. Inulin promotes the growth of beneficial gut bacteria and has various physiological functions, such as regulating blood glucose homeostasis and gastrointestinal function, and modulating the immune system. Recently, the importance of inulin has grown, not only in the food industry. It is also finding more and more applications in the medical field to alleviate diabetes and obesity (Zhou et al., 2023). Furthermore, inulin is used in the field of micro/nanotechnology to develop delivery systems for drugs, bioactive food ingredients, and live cells (Gruskiene et al., 2024).

In this study, a new multilayer delivery system for CAR is presented. For the first time, β-carotene-inulin complexes coated with lactoferrin have been developed. FT-IR and Raman spectroscopy, dynamic light scattering, fluorescence, and rheological measurements demonstrate the interaction of the components. The fabricated particles are dispersible in water. This property enhances the possibilities for CAR applications. Furthermore, the new CAR complexes combine the health-promoting properties of their components and can be an effective additive in functional foods, particularly water-based ones.

## 2 Materials and methods

### 2.1 Materials

Bovine lactoferrin (96% purity; iron content 12 mg/100 g) was purchased from Lactoferrin Co (Australia). Inulin from dahlia tubers, Trolox, β-carotene (≥ 93%, UV), tripyridyltriazine (TPTZ), and 2,2-diphenyl-1-picrylhydrazyl (DPPH) were from Sigma-Aldrich.

### 2.2 Preparation of β-carotene-loaded inulin-lactoferrin particles

First, β-carotene-inulin complexes (CAR-IN) were fabricated as previously described by Gabriunaite et al. (2025). Briefly, 20 mL of a 0.2 mg/mL CAR solution in acetone was added dropwise to 10 mL of 8 mg/mL of hot inulin solution (67 °C) to obtain a CAR/IN (*w*/w) ratio of 1:20. The final solution was stirred to allow immediate acetone evaporation. The solution was then filtered through 5 μm PES filters and frozen at −75 °C. Subsequently, it was freeze-dried at a pressure of 0.2 mbar using a Telstar LyoQuest freeze dryer. The resulting dry complex was stored at −20 °C. To obtain β-carotene-loaded inulin-lactoferrin (CAR-IN-LF) particles, an aqueous solution of lactoferrin (LF) in the range of 0.02–0.8 mg/mL was added dropwise to a 5 mg/mL CAR-IN solution by stirring to obtain different [CAR-IN]/[LF] ratios (w/w) of 1:0.0005, 1:0.001, 1:0.005, 1:0.01 and 1:0.1. The pH value was then adjusted to 4, 6, or 8 using NaOH or HCl. The solution was then shaken overnight at 4 °C and 850 rpm. The samples were then freeze-dried or immediately used for further investigations. The same procedure was performed with a control sample without lactoferrin. The yield was calculated as follows:

yield (%) = (m_CAR-IN-LF_/(m_CAR-IN_ + m_LF_)) × 100

where m_CAR-IN-LF_ is the amount (mg) of the CAR-IN-LF complex after freeze-drying, m_CAR-IN_ is the amount (mg) of the CAR-IN complex taken for the preparation of the CAR-IN-LF particles, and m_LF_ is the amount (mg) of LF used in the experiment.

### 2.3 Characteristics of β-carotene-loaded inulin-lactoferrin particles

To determine the solubility of the CAR-IN-LF particles, approximately 25 mg of the CAR-IN-LF complex were weighed in an Eppendorf tube, dissolved in 0.5 mL of deionised water and shaken at 850 rpm for 30 min, then the samples were centrifuged at 6000 ×*g* for 10 min. An Eppendorf Concentrator Plus centrifuge was used to dry the remaining insoluble complex. Subsequently, it was weighed and the obtained weight was used to calculate the concentration of the dissol*v*ed sample.

The hydrodynamic diameter, zeta potential (Z_p_) and polydispersity index (PDI) were measured using the dynamic light scattering method (DLS) as previously described (Gabriunaite et al., 2025).

Scanning electron microscopy (SEM) was performed using a Quattro S4 high-resolution scanning electron microscope (Thermo Fisher Scientific). The samples of particles were diluted with water, dropped onto a silicon wafer and freeze-dried. SEM images were obtained at an acceleration voltage of 2 kV.

The DPPH assay was used to determine antioxidant activity (Xiao et al., 2020). First, a 0.1 mM DPPH solution in ethanol was prepared and stirred for 1 h. Then the freshly prepared solutions of DPPH and CAR-IN-LF particles were mixed in a 1:1 (*v*/v) ratio and incubated for 2 h at 18 °C in the dark. Finally, the absorbance at 517 nm was measured in a 96-well plate. The radical scavenging activity (RSA, %) was calculated as pre*v*iously described (Yuan et al., 2013). The concentration of the sample that is required to reduce the initial concentration of DPPH by 50% (IC_50_) was calculated using the linear dependence of RSA (%) on the concentration of the sample. The Trolox equivalent antioxidant capacity (TEAC, mg TE/g) was measured as previously described (Xiao et al., 2020). To construct the calibration curve, the Trolox concentration was in the range of 0.00025–0.05 mg/mL.

The ferric reducing/antioxidant power (FRAP) of the samples was determined as described by Xiao et al. (2020). For the analysis of the sample, a FRAP reagent was used and prepared by mixing a 10 mM tripyridyltriazine (TPTZ) solution in 40 mM HCl, a 20 mM FeCl_3_ solution in water and a 0.3 M acetic acid buffer solution at pH 3.6 in a ratio of 1:1:10 (*v*/v/v), respectively. The prepared FRAP reagent was preheated to 37 °C and 180 μL were mixed with 20 μL of the sample. The mixture was then incubated in the dark at 37 °C for 30 min. Subsequently, the absorbance was recorded at 593 nm and TEAC (mg TE/g) was calculated as described in the previously published paper (Gabriunaite et al., 2025).

### 2.4 Spectroscopic analysis of β-carotene-loaded inulin-lactoferrin particles

Before registration of the FT-IR and Raman spectra, the solutions of the LF, CAR-IN, and CAR-IN-LF samples prepared at pH 6 were lyophilised.

A PerkinElmer Frontier 65 spectrometer equipped with a Universal ATR Sampling Accessory was used for FT-IR spectra recording as follows: 50 scans on average, a scan range of 800–4000 cm^−1^, and a resolution of 2 cm^−1^.

A PerkinElmer Raman Station 400 F spectrometer was used for the registration of Raman spectra. For the excitation, a near-infrared diode laser at 785 nm was applied. The laser power was 100 mV and the exposure time was less than 1.0 s.

For fluorescence experiments, an AMINCO-Bowman luminescence spectrometer (Thermo Fisher Scientific, USA) was used. Fluorescence spectra were recorded at 20 °C. The spectrometer was equipped with a constant-temperature cuvette holder. To maintain the temperature, an external constant-temperature circulator was used. The path length of the quartz cuvette was 1 cm. The wavelengths of 280 and 295 nm were applied for excitation, and emission spectra were recorded within the wavelength range of 305–450 nm. The slit widths and scan speed were 16 nm and 1 nm/s, respectively. For fluorescence measurements, the particles were obtained at pH 6, using solutions of 5 mg/mL CAR-IN and 0.5 mg/mL lactoferrin. The particle emission spectra were then compared with the 0.5 mg/mL lactoferrin spectra.

### 2.5 Rheological measurements

A CR302 rheometer (Anton Paar Ltd., Austria) equipped with a parallel plate system was used to perform rheological measurements. A plate with a diameter of 50 mm and a round disc with a gap of 2 mm were used. For rotational tests, the shear rate ranged from 1.0 s^−1^ to 100 s^−1^. Solutions of pure inulin at 4.5 mg/mL and complex IN-LF at 5 mg/mL were prepared at three pH values of 4, 6, and 8. The [IN]/[LF] ratio (*w*/w) of 1:0.1 was used for the complex preparation. The rheological experiments were performed at room temperature.

### 2.6 Incorporation of β-carotene-loaded inulin-lactoferrin particles into foods

The lyophilised CAR-IN-LF particles fabricated in a [CAR-IN]/[LF] ratio (w/w) of 1:0.1 at pH 6 were dissolved in birch sap and mineral water to a final concentration of 1 mg/mL. Particles were also added to natural yogurt at a concentration of 3.8 mg per 1 g yogurt. The fortified beverages were stored in the dark at 4 °C and 20 °C and in light at 20 °C. The fortified yogurt was stored at 4 °C in the dark. The colour parameters in the CIELAB colour space were measured over a period of one month using a Konica Minolta CM-700d spectrophotometer. For the calibration of the spectrophotometer, a white background was used, and then the L*, a* and b* parameters, which describe the lightness, green-red and blue-yellow colours, respectively, were recorded. Subsequently, the colour saturation or chroma (C*) and the total colour difference (ΔE) were calculated (Pathare et al., 2013):

$$C^{*}=\sqrt{{a^{*}}^{2}+{b^{*}}^{2}}$$

$$∆E^{*}=\sqrt{∆{L^{*}}^{2}+∆{a^{*}}^{2}+∆{b^{*}}^{2}}$$

The reaction rate constants (k) and the half-lives (t₁/₂) were calculated by fitting the dependence of C* and ΔE on time according to the kinetics of the first-order reaction.

### 2.7 Statistical analysis

Data were presented as mean ± standard deviation. To define statistically significant results, a one-way ANOVA (*P* < 0.05) was used. For that, three independent experiments were performed.

## 3 Results and discussion

### 3.1 Fabrication and characterisation of the ternary β-carotene-loaded particles

**Fig. 1:**
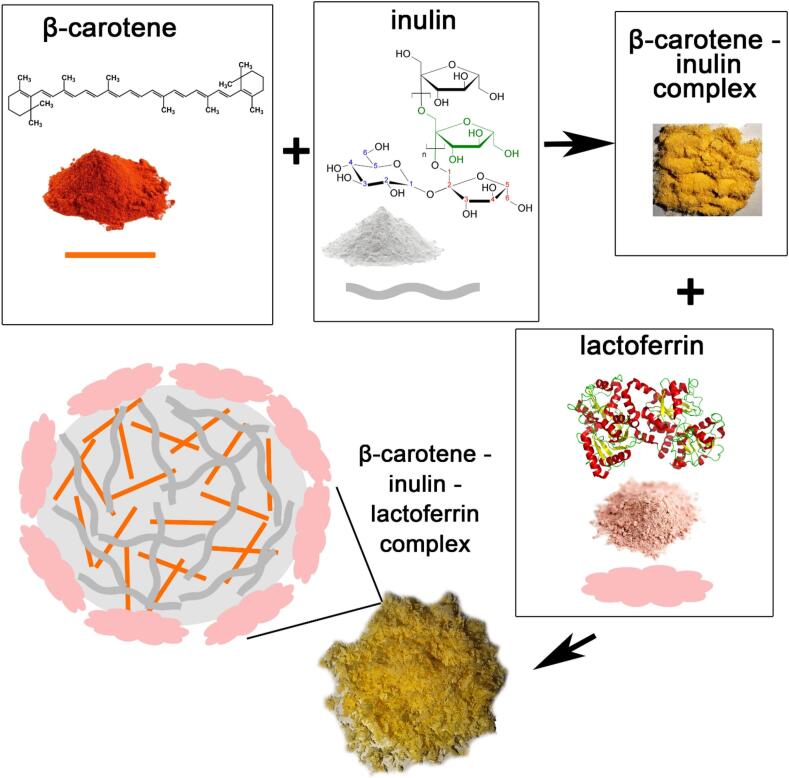
Scheme of preparation of β-carotene-loaded inulin-lactoferrin particles.

**Fig. 2:**
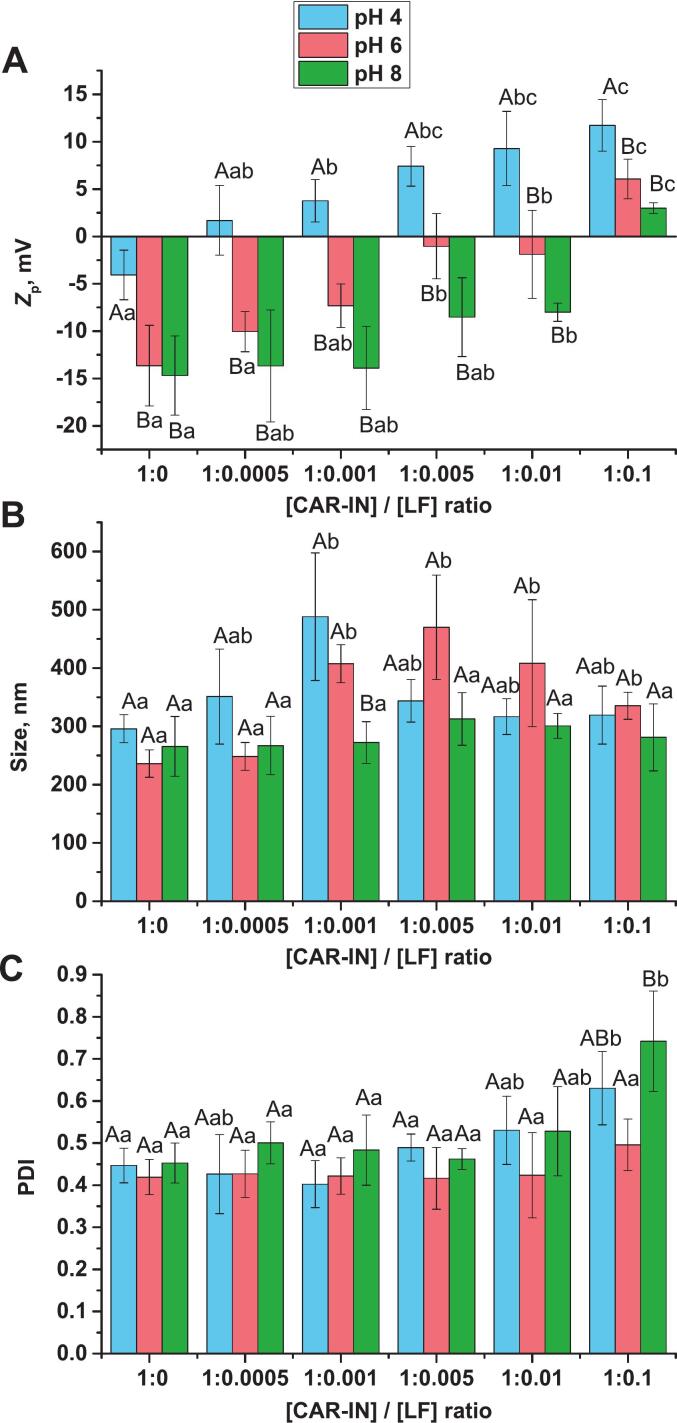
Characteristics of β-carotene-loaded inulin-lactoferrin particles fabricated at different pH values and in different [CAR-IN]/[LF] ratios; (A), zeta-potential; (B), size; (C), polydispersity index. Different uppercase letters show significant differences in the mean of the parameters in the same [CAR-IN]/[LF] ratio; *P* < 0.05. Different lowercase letters show significant differences in the mean of the parameters at the same pH value, but in a different [CAR-IN]/[LF] ratio; P < 0.05.

**Fig. 3:**
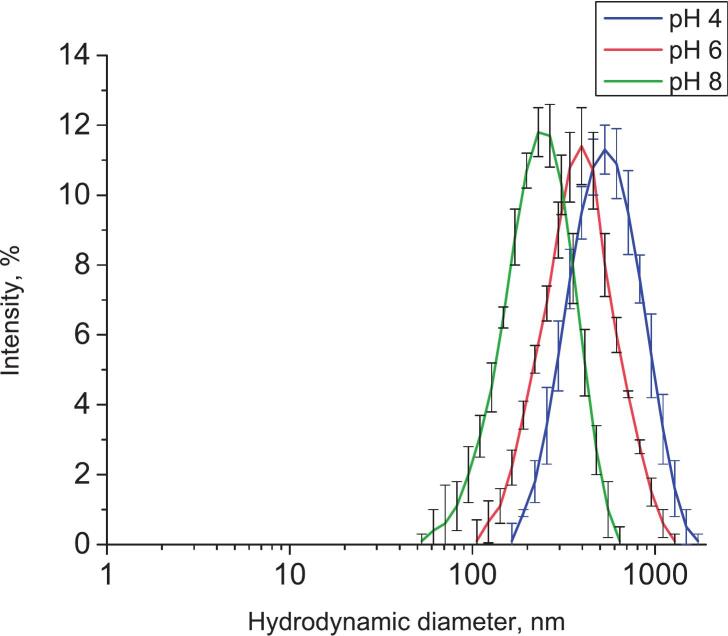
Size distribution of β-carotene-loaded inulin-lactoferrin particles fabricated in a [CAR-IN]/[LF] ratio of 1:0.01.

**Fig. 4:**
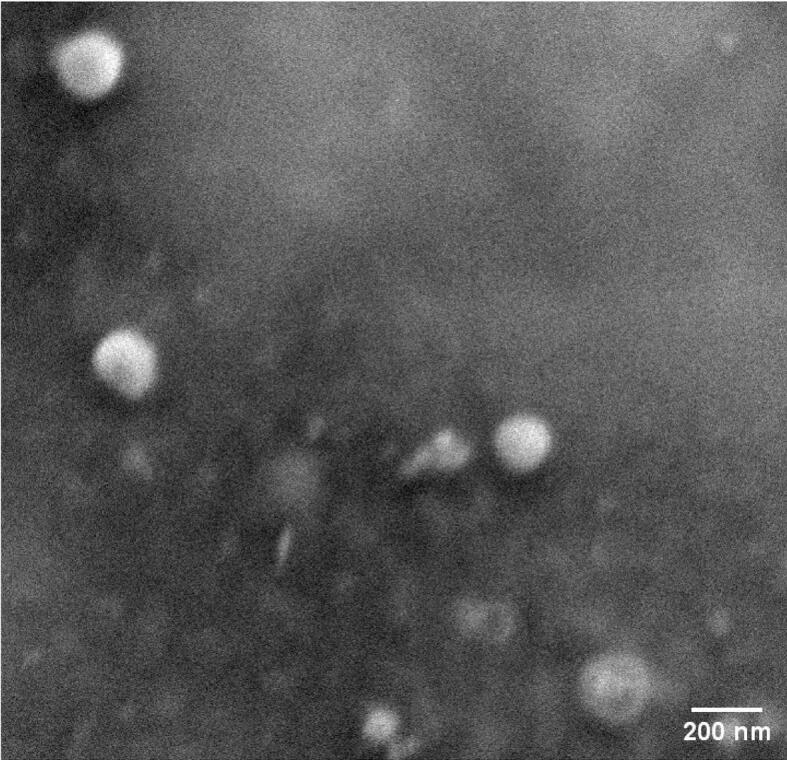
SEM image of CAR-IN-LF particles.

The ternary β-carotene loaded system was prepared in two steps as illustrated in Fig. 1. First, the CAR-IN particles were fabricated as previously published (Gabriunaite et al., 2025). Secondly, the CAR-IN particles were coated with lactoferrin using an increasing amount of the protein. This coating process was performed at different pH values. As can be seen in Fig. 2A, the lower the pH value, the smaller the amount of lactoferrin required to reduce the negative surface charge of the particles. At pH 4, a [CAR-IN]/[LF] ratio (*w*/w) of 1:0.0005 is sufficient for the particle zeta-potential to transit from negative to positive. Inulin is generally known as a non-ionic polysaccharide (Mensink et al., 2015). However, several previous studies have shown that the zeta-potential of inulin in solution is negative. This surface charge can be caused by hydroxyl groups in inulin molecules (Lopez-Castejon et al., 2019; 2021; Krivorotova et al., 2016; Mandracchia et al., 2014). On the other hand, many colloidal dispersions in water have a negative surface charge, supposedly due to the preferential adsorption of hydroxyl ions (Hornig et al., 2009; Marinova et al., 1996). Our study shows that the negative surface charge of the CAR-IN particles decreases with a decrease in the pH value of the solution (Fig. 2A). The isoelectric point of bovine lactoferrin is in the basic pH region between 9.3 and 9.5 (Voswinkel et al., 2016). Therefore, electrostatic interactions between the negatively charged CAR-IN particles and the positively charged lactoferrin promote the formation of ternary particles. The encapsulation yield and solubility of the complexes practically depended insignificantly on the [CAR-IN]/[LF] ratio. Only in a 1:0.1 ratio, the obtained ternary complex was slightly more soluble than in other ratios (Table 1). The hydrodynamic diameter of the particles was 250–300 nm or greater depending on the [CAR-IN]/[LF] ratio and the pH value (Fig. 2B). At pH 4 and 6, the size showed a tendency to increase when the amount of LF used for coating increased. However, this effect was not observed at pH 8. The surface charge of the CAR-IN particles differs at different pH values. Furthermore, lactoferrin becomes less positive at higher pH values near its isoelectric point. In addition to electrostatic forces, hydrogen bonding and hydrophobic interactions can affect the formation of the ternary complex (Li et al., 2023; Meng et al., 2024). Furthermore, the contribution of each type of force to the lactoferrin-inulin interaction may differ at different pH values. Taking it all together, the effect of the amount of lactoferrin on its arrangement on the CAR-IN surface and the size of the complex could differ at different pH values. The polydispersity index was higher than 0.4 for uncoated and coated with lactoferrin particles independently of the pH value and showed a tendency to increase in the [CAR-IN]/[LF] ratio of 1:0.1 (Fig. 2C). The obtained β-carotene-loaded ternary particles can be qualified as broadly polydisperse (Bhattacharjee, 2016). The example of particle size distribution is presented in Fig. 3. As shown by scanning electron microscopy, the particles had almost spherical shape (Fig. 4).

**Table 1:** Preparation of β-carotene-loaded inulin-lactoferrin particles in different [CAR-IN]/[LF] ratios.

[CAR-IN]/[LF] ratio, *w*/w	Yield, %	Solubility, mg/mL
1:0	58.8 ± 12.4 ^a^	20.8 ± 1.7 ^ab^
1:0.0005	52.6 ± 5.2 ^a^	17.8 ± 2.3 ^b^
1:0.001	57.9 ± 10.8 ^a^	18.0 ± 0.3 ^b^
1:0.005	59.1 ± 9.6 ^a^	18.0 ± 0.9 ^b^
1:0.01	54.0 ± 7.2 ^a^	17.4 ± 4.7 ^ab^
1:0.1	62.5 ± 10.5 ^a^	22.9 ± 2.5 ^a^

### 3.2 Spectroscopic and rheological analysis of the particle component interaction

**Fig. 5:**
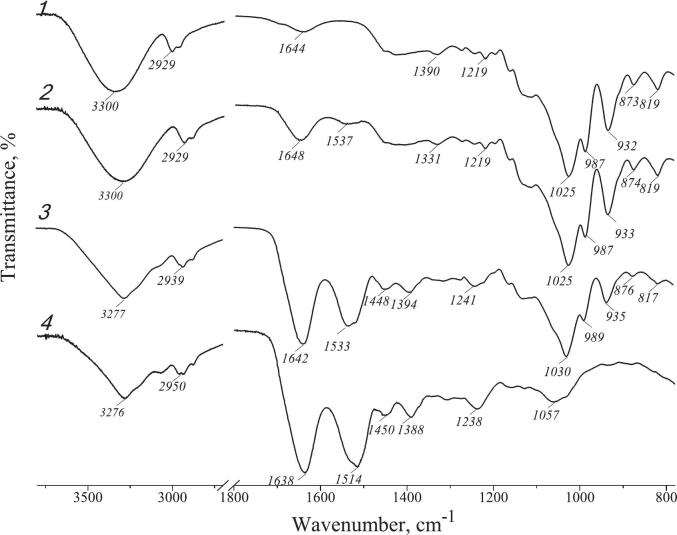
FT-IR spectra of inulin (1), CAR-IN-LF particles fabricated in a [CAR-IN]/[LF] ratio of 1:0.1 (2) and 1:1 (3) and lactoferrin (4).

FTIR spectra of the inulin, lactoferrin and lactoferrin-coated CAR-IN particles are demonstrated in Fig. 5. The bands at 3300 cm^−1^ and 2929 cm^−1^, seen in the inulin spectrum, are assigned to O—H and C—H stretching vibrations, respectively. The absorption bands at 1025 cm^−1^ and 987 cm^−1^ are attributed to C—O stretching vibrations in the furanose ring. The presence of β-(2 → 1) linkage in inulin is indicated by the band at 933 cm^−1^. The band at 1644 cm^−1^ is due to water absorption (Barclay et al., 2016; Grube et al., 2002; Maqbool et al., 2025). In the lactoferrin spectrum, the bands of Amide *I* and II are at 1638 cm^−1^ and 1514 cm^−1^, respectively (Fig. 4, curve 4) as previously reported (Lin et al., 2022). The spectrum of CAR-IN-LF particles obtained in a [CAR-IN]/[LF] ratio of 1:0.1 shows that the bands of Amide *I* and II shift to the higher wavenumbers, to 1648 cm-1 and 1537 cm-1, respectively (Fig. 5, curve 2). The region of Amide *I* and II is sensitive to secondary structure and conformational changes (Barth & Zscherp, 2002) and indicates the interaction between lactoferrin and particle inulin. In the case of CAR-IN-LF particles obtained in a [CAR-IN]/[LF] ratio of 1:1, the shift is also observed but is less pronounced. It is plausible that some free lactoferrin was present in the sample because the DLS experiments showed that the positive particle zeta-potential increased up to a [CAR-IN]/[LF] ratio of 1:0.25.

**Fig. 6:**
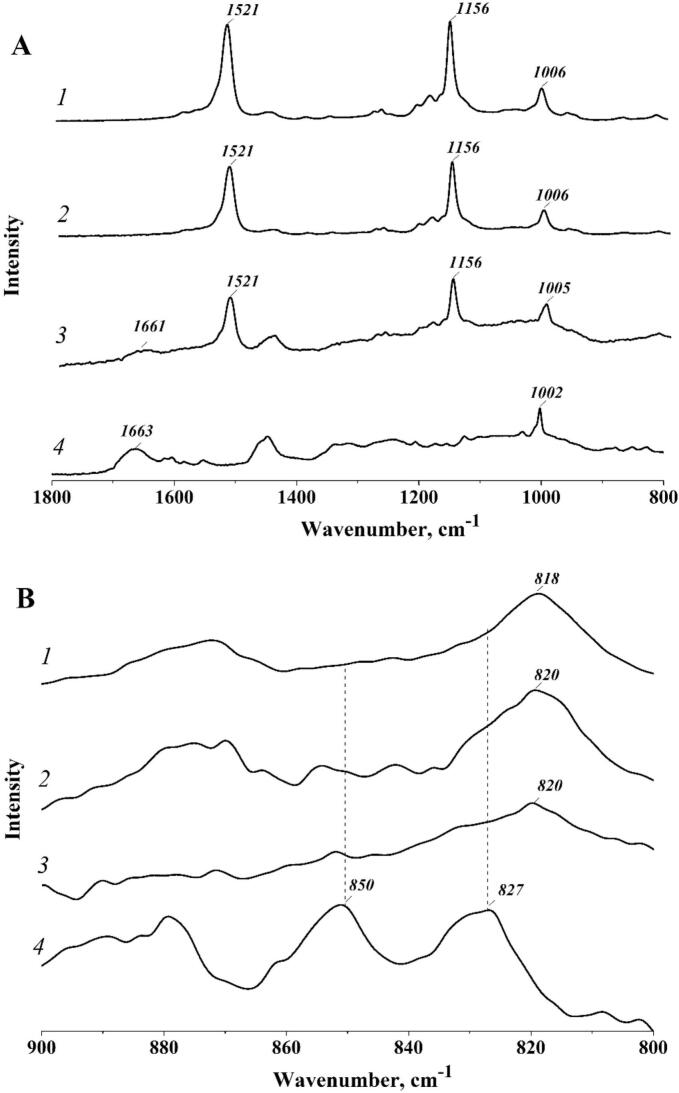
Raman spectra (A, 1800–800 cm^−1^ and B, 900–800 cm^−1^) of CAR-IN particles (1), CAR-IN-LF particles fabricated in a [CAR-IN]/[LF] ratio of 1:0.1 (2) and 1:1 (3) and lactoferrin (4).

Fig. 6 shows the Raman spectra of uncoated and lactoferrin-coated CAR-IN particles. Our previous research demonstrated that in the CAR-IN spectra, the ν1 band of β-carotene at 1512 cm-1 corresponded to the C=C stretching vibration of the single polyene molecule. It became broader and shifted to higher wavenumbers (Gabriunaite et al., 2025). This indicates conformational changes and a partial loss of double bond conjugation in β-carotene due to its complexation with polysaccharides (Macernis et al., 2021; Straksys et al., 2022). In the case of lactoferrin-coated CAR-IN particles, no further shift of the ν_1_ band was observed (Fig. 6A, curves 2 and 3) and found at 1521 cm^−1^ as for CAR-IN particles (Fig. 6A, curve 1). This means that lactoferrin does not interact with β-carotene due to the inulin matrix. The Raman spectrum of lactoferrin (Fig. 6A, curve 4) exhibits the characteristic band of Amide *I* at 1663 cm^−1^ (Iafisco et al., 2011). Unfortunately, this band is not obviously seen in the spectrum of CAR-IN-LF particles (Fig. 6A, curves 2 and 3). The Raman spectrum of lactoferrin in the region of 900–800 cm^−1^ (Fig. 65B, curve 4) shows the tyrosine doublet at 850/827 cm^−1^, which provides information about the hydrogen bonding of the phenolic hydroxyl group (Li-Chan, 2007). The intensity of the tyrosine doublet decreases markedly in the Raman spectra of CAR-IN-LF particles (Fig. 6B, curves 2 and 3). This suggests an interaction between lactoferrin and CAR-IN particles, resulting in changes in the tyrosine environment due to alterations in the protein structure (Zhang et al., 2012).

**Fig. 7:**
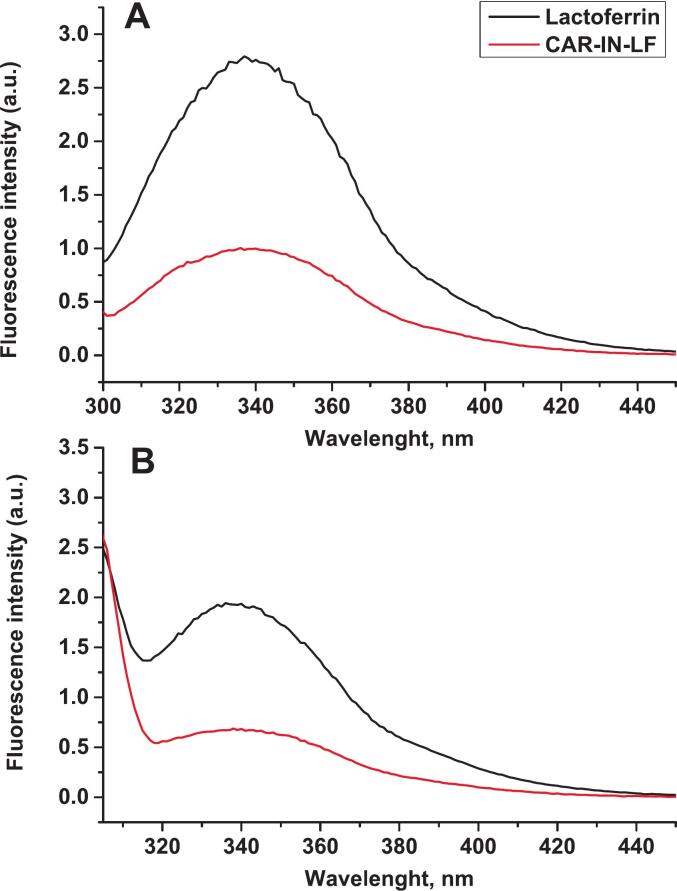
Fluorescence emission spectra of the lactoferrin and CAR-IN-LF complex at 280 nm (A) and 295 nm (B) excitation. The concentration of lactoferrin in both samples was the same. The [CAR-IN]/[LF] complex was obtained in a [CAR-IN]/[LF] ratio of 1:0.1 and pH 6.

To demonstrate the interaction between lactoferrin and CAR-IN particles, spectrofluorimetric measurements were performed. Fig. 7 shows the fluorescence spectra of free lactoferrin and lactoferrin complexed with CAR-IN particles, registered at excitation wavelengths of 280 nm and 295 nm. Lactoferrin tryptophan is excited at 295 nm, while tryptophan and tyrosine are excited at 280 nm. Both lactoferrin emission spectra are similar to those previously reported (Jing et al., 2021). In the case of coated particle spectra, the reduction in fluorescence intensity suggests interaction between the CAR-IN and lactoferrin. However, the λ_max_ values of the emission spectra did not change at both excitation wavelengths. The interaction of lactoferrin with the okra polysaccharide or small molecule tetracycline hydrochloride also did not change the maximum wavelength of the emission, suggesting that the fluorophore environment is not altered (Sun et al., 2018; Xu et al., 2019).

**Fig. 8:**
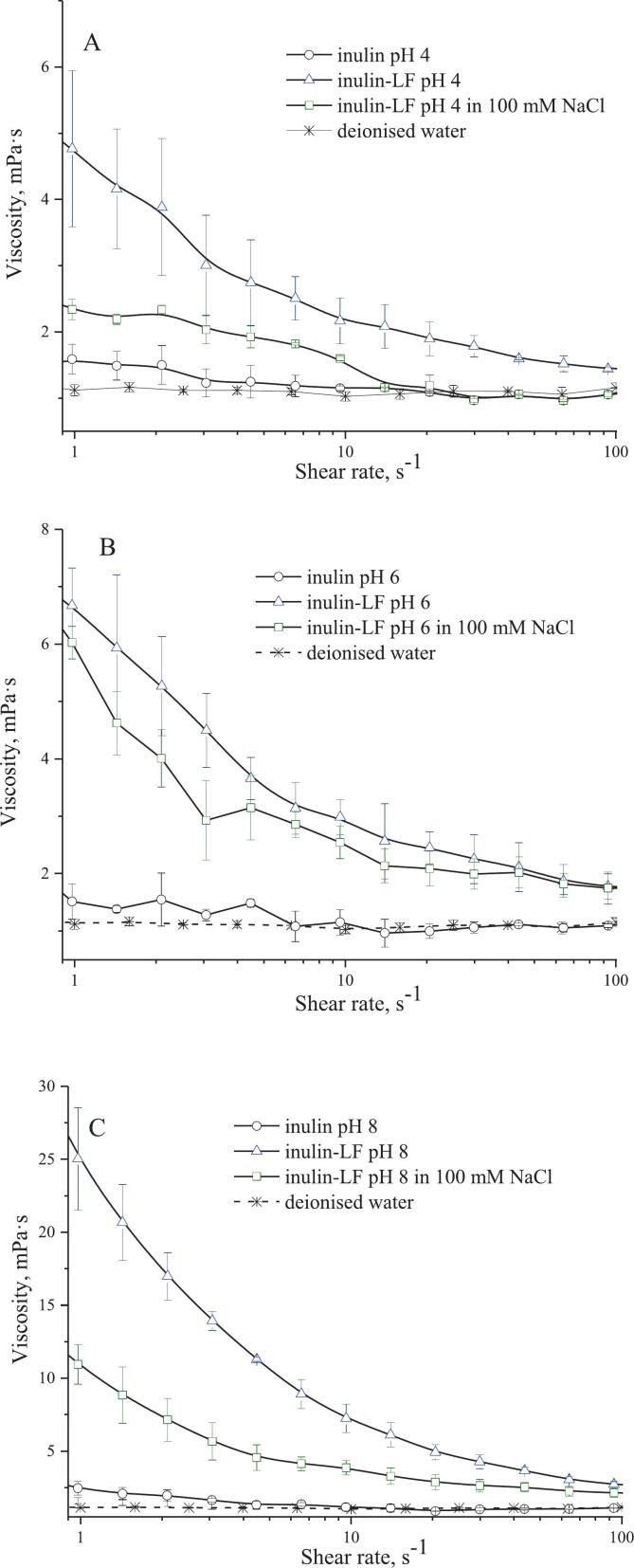
The flow behaviour of solutions of pure inulin at a concentration of 4.5 mg/mL and complex inulin-LF at a concentration of 5 mg/mL in the presence and absence of 100 mM NaCl; pH 4 (A), 6 (B) and 8 (C). The [IN]/[LF] ratio was 1:0.1 (*w*/w).

In addition, to demonstrate the possible interaction between inulin and lactoferrin, rheological measurements were performed at different pH values in the absence and in the presence of 100 mM NaCl. As can be seen in Fig. 8, the flow curves of the aqueous inulin solutions at pH 4, 6, and 8 exhibited Newtonian behaviour, with no change in viscosity observed over the shear rate of 1 s^−1^ to 100 s^−1^. However, the addition of lactoferrin resulted in the shear-thinning effect of IN-LF solutions (Fig. 8). This effect generally occurs in electrostatic complexes of protein-polysaccharide (Liu et al., 2017; Zheng et al., 2023). The addition of 100 mM NaCl indeed reduced viscosity due to a decrease in ionic-strength-dependent interactions between inulin with the negative surface charge (Gabriunaite et al., 2025; Krivorotova et al., 2016) and cationic lactoferrin (Voswinkel et al., 2016). These rheological measurements indirectly demonstrate the possibility of an interaction based on electrostatic neutralisation of opposite charges between the β-carotene-loaded inulin particles and the lactoferrin used for coating.

### 3.3 Antioxidant activity of the β-carotene-loaded inulin-lactoferrin particles

**Fig. 9:**
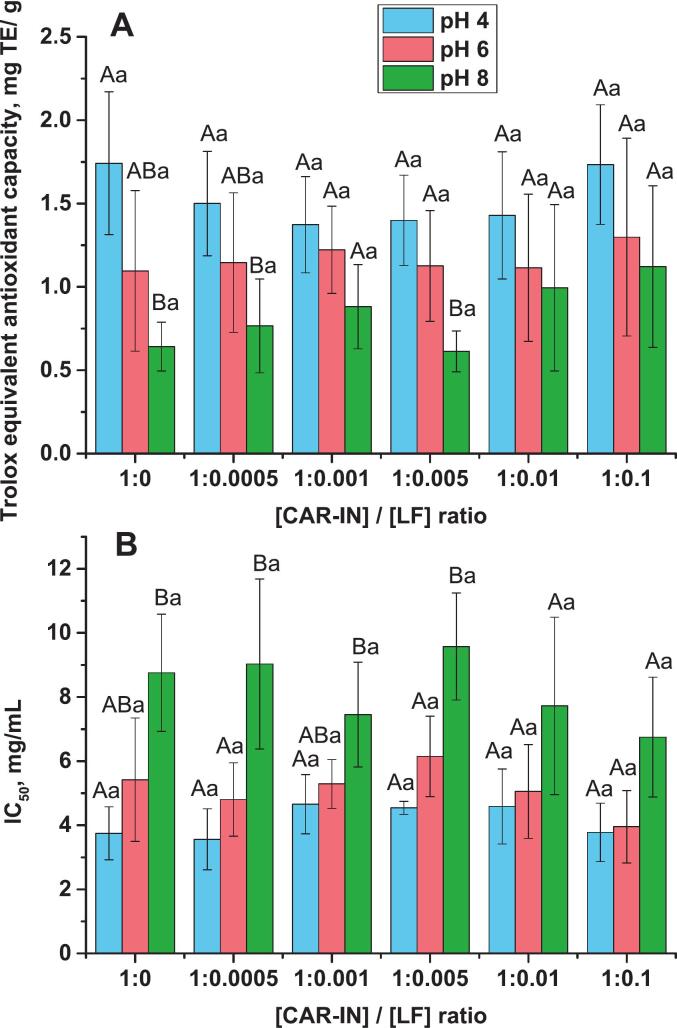
DPPH antioxidant activity of the particles in Trolox equivalents (A) and the concentration of the CAR-IN-LF complex that reduces the initial concentration of DPPH by 50% (B).

**Fig. 10:**
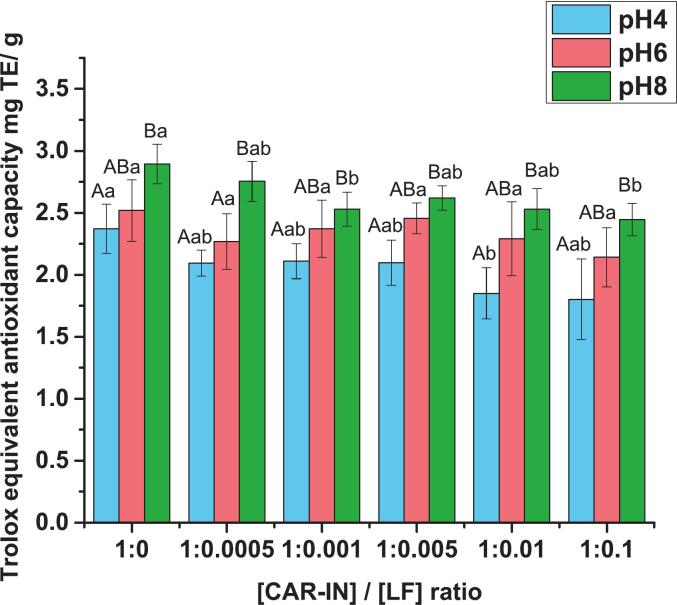
FRAP antioxidant activity of the particles in Trolox equivalents. Different uppercase letters show significant differences in the mean of the parameters in the same [CAR-IN]/[LF] ratio; P < 0.05. Different lowercase letters show significant differences in the mean of the parameters at the same pH value, but in a different [CAR-IN]/[LF] ratio; P < 0.05.

The antioxidant activity of the fabricated particles was evaluated by measuring their capacity to scavenge DPPH radicals and their ferric reducing power, using the FRAP method (Fig. 9, Fig. 10). As can be seen, the radical scavenging activity and IC_50_ of the particles uncoated and coated with a low amount of lactoferrin depended on the pH values at which they were fabricated. As inulin antioxidant activity is low (Shang et al., 2018) and the mechanism by which inulin scavenges radicals is not fully clear (Wang et al., 2016), β-carotene acts as the main electron donor to the DPPH radical (Sandmann, 2019). It is plausible that, at higher pH values, the protection provided by inulin against β-carotene degradation is insufficient, resulting in lower registered radical scavenging activity. A higher amount of lactoferrin masks these differences and provides better protection for β-carotene. Furthermore, lactoferrin itself has antioxidant activity (Gruden et al., 2026). The presence of amino acids such as methionine and cysteine in the structure of lactoferrin contributes to its antioxidant activity (Wang et al., 2024; He et al., 2025). Additionally, aromatic amino acid residues of lactoferrin can donate protons to stable DPPH radicals (Zhang et al., 2022; Zilic et al., 2012). This also justifies the usefulness of additional coating of particles. The fabricated particles also demonstrated the ability to reduce the Fe^3+^/tripyridyltriazine complex to Fe^2+^/tripyridyltriazine in the FRAP assay (Fig. 10). The pH value at which the particles are obtained affects the conformation of the layer molecules and their interactions with each other and with loaded bioactive molecules. Sreedhara et al. (2010) demonstrated that bovine lactoferrin is structurally stable at pH 5–8, undergoing conformational changes at pH below 5. The sum of various factors influences the electron extraction and antioxidant capacities of the particles.

In summary, the antioxidant activity of β-carotene encapsulated in newly prepared particles was retained, and these particles could be used as antioxidants in functional foods. Previously published papers have also demonstrated the antioxidant activity of carotenoids when they are complexed with polysaccharides (Bockuviene et al., 2019; 2021; Straksys et al., 2024).

### 3.4 β-Carotene-loaded inulin-lactoferrin particles for fortifying beverages and yogurt

**Fig. 11:**
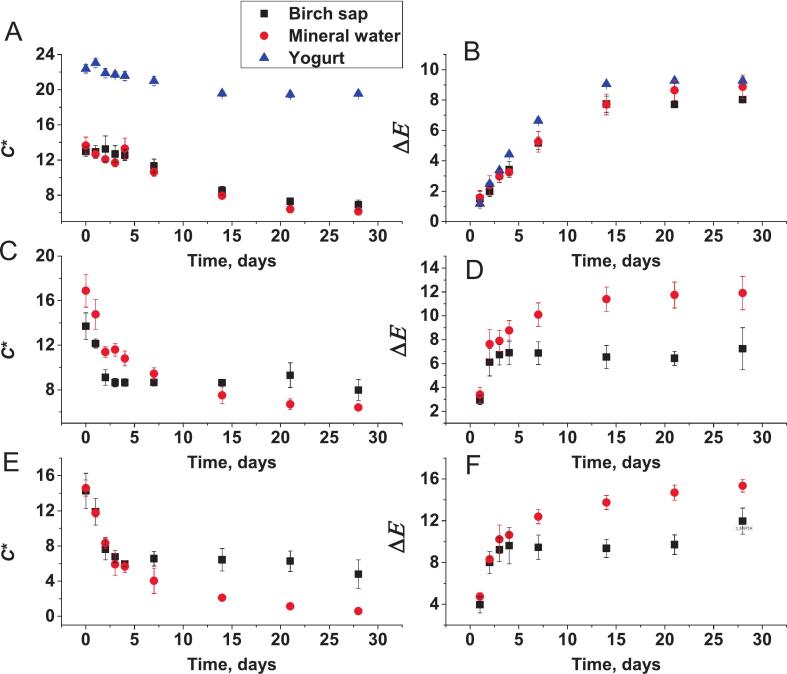
Changes in chroma (C*) and total colour difference (Δ*E*) in birch sap, mineral water, and natural yogurt samples fortified with CAR-IN-LF particles stored at 4 °C in the dark (A, B), 20 °C in the dark (C, D) and 20 °C in the light (E, F) for 28 days.

**Fig. 12:**
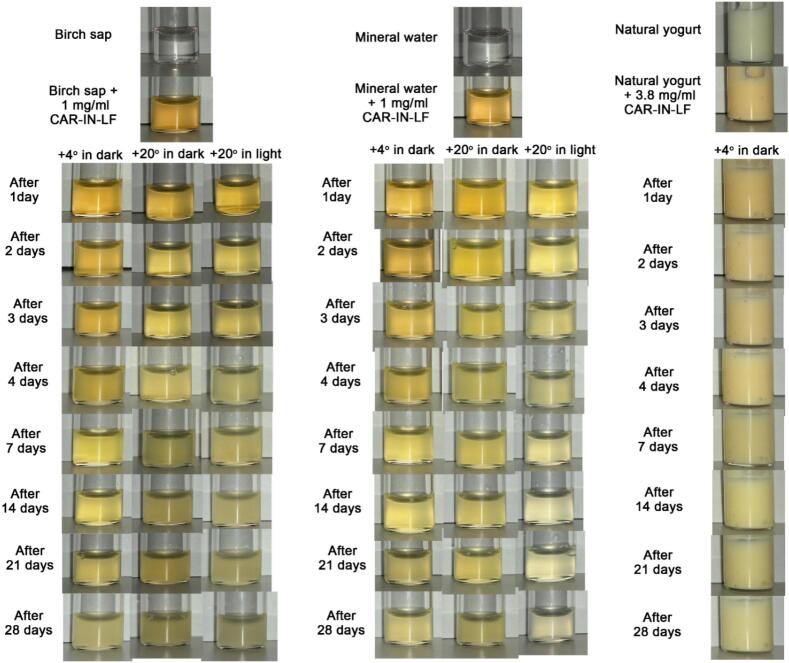
Photographs of samples of fortified birch sap, mineral water, and natural yogurt with CAR-IN-LF particles. The final concentration of CAR-IN-LF particles was 1 mg/mL in birch sap and mineral water, and 3.8 mg/mL in natural yogurt. The samples were kept for 4 weeks under different conditions.

To test the application of prepared CAR-IN-LF particles for fortifying foods and beverages, they were incorporated into three different products: store-bought birch sap, a common drink in the Baltic region obtained from birch tree, store-bought mineral water, and natural yogurt. The pH of store-bought birch sap was measured to be 4.40 ± 0.05; however, the pH value of freshly obtained birch sap can be between 5.5 and 7.5 depending on seasonal variations (Kallio & Ahtonen, 1987). In this case, the lower pH is most likely due to the addition of citric acid as a preservative and flavour enhancer during the bottling process. The pH of store-bought mineral water was 7.8 ± 0.1. The water was obtained from a spring in Lithuania and contained various minerals, with a total concentration of dissolved substances of 1378 mg/L. The pH of the natural yogurt was 4.70 ± 0.01. Therefore, acidic and alkaline foods were tested for incorporation of CAR-IN-LF particles. The amount of CAR-IN-LF to be incorporated into foods was chosen based on the recommendations of the German Federal Institute for Risk Assessment (Bundesinstitut für Risikobewertung, 2021). A maximum of 0.45 mg/100 mL of β-carotene is allowed to be incorporated into beverages and 1.7 mg/100 g into solid foods. This would correspond to 1 mg/mL of CAR-IN-LF in beverages (birch sap and mineral water) and 3.8 mg/g of CAR-IN-LF in solid foods (yogurt). Fig. 11 shows the changes in C* and Δ*E* of beverages and yogurt fortified with the CAR-IN-LF complex under different storage conditions. Table 2 summarises the kinetic parameters of C* and Δ*E*. As expected, the slowest changes in the colour parameters occurred when the products were stored in the dark at 4 °C. Furthermore, there were no large differences in the rate of change in the colour parameters depending on the food matrix (Table 2). After one week of storage at 4 °C, Δ*E* of yogurt, birch sap and mineral water were 6.6, 5.0 and 5.3, respectively (Fig. 11B). If Δ*E* is 3.0–6.0, the colour difference is clearly visible and detectable by ordinary people. When Δ*E* > 6, the colour difference is large, but not extreme (Δ*E* > 12) (Khan et al., 2014). Considering the short expiry date of natural yogurt, the results obtained are satisfactory. At 20 °C, the rate of change in colour parameters was faster for birch sap (4.5–4.8 (ΔE) and 7.1–8.6 (C*) times) and mineral water (2.3–2.7 (ΔE) and 2.6–2.9 (C*) times). In addition, photographs of the vials containing the fortified beverages and yogurt were taken using a smartphone and presented as pictures (Fig. 12). Therefore, the addition of CAR-IN-LF should be preferable to functional foods stored in the refrigerator. Furthermore, the incorporation of CAR-IN-LF particles prepared in various [CAR-IN]/[LF] ratios should be investigated for the fortification of functional beverages, which could have a more stable colour at room temperature. In addition, coencapsulation of β-carotene with other antioxidants, such as α-tocopherol, could be considered. Brito-Oliveira et al. (2017) demonstrated that α-tocopherol significantly retards the degradation of β-carotene when both compounds are coencapsulated in solid lipid microparticles.

**Table 2:** Kinetic parameters of the colour changes in beverages and yogurt fortified with CAR-IN-LF particles under various storage conditions.

Storage Conditions	Food sample	*Chroma*		*ΔE*	
		*k*, d^−1^	*t*_1/2_, d	*k*, d^−1^	*t*_1/2_, d
4°C, dark	Birch sap	0.07 ± 0.01	9.42 ± 1.14	0.16 ± 0.02	4.45 ± 0.57
	Mineral water	0.09 ± 0.02	7.83 ± 1.6	0.14 ± 0.01	5.13 ± 0.47
	Natural yogurt	0.11 ± 0.01	6.15 ± 0.68	0.17 ± 0.01	4.08 ± 0.04
					
20 °C, dark	Birch sap	0.60 ± 0.14	1.24 ± 0.38	0.77 ± 0.18	0.97 ± 0.30
	Mineral water	0.23 ± 0.03	3.04 ± 0.36	0.38 ± 0.02	1.86 ± 0.12
					
20 °C, light	Birch sap	0.50 ± 0.18	1.63 ± 0.73	0.72 ± 0.07	0.98 ± 0.09
	Mineral water	0.26 ± 0.04	2.76 ± 0.66	0.32 ± 0.02	2.19 ± 0.14

## 4 Conclusion

A new formulation of β-carotene was developed using two beneficial biopolymers for health: inulin and lactoferrin. The interaction between these components and the formation of particles was demonstrated using dynamic light scattering, fluorescence measurements, and FT-IR and Raman spectroscopy. Rheological measurements were also performed to investigate the possible interactions between inulin and lactoferrin. The antioxidant activity of the β-carotene-loaded inulin-lactoferrin particles was also demonstrated. The potential applications of the new formulation were studied in real food systems such as yogurt, mineral water, and birch sap. The samples stored at 4 °C exhibited the best colour preservation. Furthermore, the rate of change in colour did not differ significantly depending on the food matrix. To improve the stability of β-carotene in food products stored at room temperature, fortification of food products with particles fabricated in different ratios of the β-carotene-inulin complex and lactoferrin should be considered.

## CRediT authorship contribution statement

**Inga Gabriunaite:** Writing – original draft, Investigation, Conceptualization. **Tatjana Kavleiskaja:** Visualization, Investigation. **Rūta Gruškienė:** Validation, Conceptualization. **Jolanta Sereikaitė:** Writing – review & editing, Supervision, Project administration, Conceptualization.

## Funding

The research was funded by the 10.13039/501100004504Research Council of Lithuania, project No S-PD-24-55.

## Declaration of competing interest

The authors declare that they have no known competing financial interests or personal relationships that could have appeared to influence the work reported in this paper.

## Data Availability

Data will be made available on request.
